# Sustainable Aromatic Aliphatic Polyesters and Polyurethanes Prepared from Vanillin-Derived Diols via Green Catalysis

**DOI:** 10.3390/polym12030586

**Published:** 2020-03-05

**Authors:** Changbo Zhao, Caijuan Huang, Qin Chen, Ian D. V. Ingram, Xiankui Zeng, Tianhua Ren, Haibo Xie

**Affiliations:** 1 Department of Polymer Materials &Engineering, College of Materials & Metallurgy, Guizhou University, West Campus, Huaxi District, Guiyang 550025, Chinaqchen6@gzu.edu.cn (Q.C.);; 2 Department of Natural Sciences, Manchester Metropolitan University, Chester Street, Manchester M1 5DG, UK; i.ingram@mmu.ac.uk

**Keywords:** lignin, vanillin, green catalysis, full bio-based polyesters, polyurethane

## Abstract

The design and preparation of polymers by using biobased chemicals is regarded as an important strategy towards a sustainable polymer chemistry. Herein, two aromatic diols, 4-(hydroxymethyl)-2-methoxyphenol and 2-(4-(hydroxymethyl)-2-methoxyphenoxy)ethanol, have been prepared in good yields through the direct reduction of vanillin and hydroxyethylated vanillin (4-(2-hydroxyethoxy)-3-methoxybenzaldehyde) using NaBH_4_, respectively. The diols were submitted to traditional polycondensation and polyaddition with acyl chlorides and diisocyanatos, and serials of new polyesters and polyurethanes were prepared in high yields with moderate molecular weight ranging from 17,000 to 40,000 g mol^−1^. Their structures were characterized by ^1^H NMR, ^13^C NMR and FTIR, and their thermal properties were studied by TGA and differential scanning calorimetry (DSC), indicating that the as-prepared polyesters and polyurethanes have T_g_ in the range of 16.2 to 81.2 °C and 11.6 to 80.4 °C, respectively.

## 1. Introduction

With the continuous exploitation of petroleum-based resources, energy and materials, humanity faces serious challenges in terms of both climate change and the availability of useable resources [1]. In particular, the use and disposal of non-degradable plastics in our daily lives leads to serious microplastic particle accumulation and pollution in both soil and aquatic environments [2]. Therefore, it is of great importance to develop more sustainable polymer materials using renewable resources and green chemistry technologies [3].

Lignocellulosic biomass is the most abundant biomass resources on the planet with more than 75 billion tons available per year [4], and the use of them to produce energy, chemicals and materials is anticipated to reduce our dependence on fossil resources, contributing to development of bio-economy [5,6]. Lignocellulosic biomass is composed of cellulose, hemicelluloses and lignin, in which lignin is mainly composed of aromatic units and is the most abundant natural aromatic hydrocarbon on earth [7,8]. In recent years, there has been significant interest in the catalytic conversion of lignocellulose to bio-based platform molecules [9,10]. For example, cellulose can be converted to methanol, ethanol, ethylene glycol, glucose and 5-hydroxymethylfurfural [11]. A variety of aromatic chemicals can be obtained by catalytic conversion of lignin, including eugenol, vanillin, p-hydroxybenzaldehyde and terephthalic acid [1,12]. Traditionally, vanillin is one of the most important perfume compounds in foods, beverages, perfumes and pharmaceuticals and is originally isolated from vanilla plants [13,14]. Vanillin can also be produced by various biotechnological approaches and catalytic conversion of lignin, for example, using lignin-derived ferulic acid and glucose as substrates for fermentation with bacteria, fungi or yeasts [13]. Given its useful structural features and the potential for its large-scale production from lignin, vanillin is considered to be a promising bio-based building block chemical for the preparation of sustainable aromatic polymers [15,16]. Vanillin derivatives with desirable functionalities for polymer synthesis can be obtained through carboxylation, hydroxylation, olefination and amination [17]. Vanillin can be converted to acetyl dihydroferulic acid, which can itself be polymerized to poly(dihydroferulic acid), having similar thermal properties to PET [18]. Meylemans et al. hydrogenated vanillin to prepare 2-methoxy-4-methylphenol, which was condensed with an aldehyde under acid catalysis to give a substitute of bisphenol A, with potential utility in epoxy resins and polyurethanes [19]. Bai et al. used vanillin and triphosgene to prepare a dialdehyde monomer having a carbonate structure, which can be reduced to the corresponding diol, offering great opportunities to prepare novel polyesters and polyurethanes incorporating a carbonate moiety in the main chain [20]. Zhu et al. prepared a bisphenol structure via a one-pot method using vanillin, diamine and diethyl phosphite, which was then reacted with epichlorohydrin to synthesize a novel bio-based epoxy resin with excellent flame retardancy, as well as good thermal and mechanical properties [21]. Based on this idea, Zhu et al. prepared a vanillin derived mono-epoxide, which could react with a diamine to form a recyclable and high performance thermosetting resin containing a Schiff base structure, and then studied its application in carbon fiber composites [22]. Llevot et al. prepared both a biphenyl bisphenol monomer and a biphenyl esterified monomer using vanillin, and synthesized a series of amorphous thermoplastic polyesters with thermal stability up to 350 °C by transesterification, showcasing the possibilities of new symmetrical bio-based monomers [23]. Firdaus et al. used a vanillin derived monomer to prepare a series of polymers with molecular weights up to 50 KDa by thiol-ene addition, polycondensation and ADMET polymerization [24]. Wang et al. obtained a resin with a T_g_ of up to 184 °C and a Young’s modulus and hardness higher than those of conventional bisphenol A epoxy resins [25]. Tao et al. introduced a trifluorovinyl ether group in vanillin and polymerized the methoxy group in vanillin with disiloxane by the Piers-Rubinsztajn reaction, obtaining a novel fluoro-containing polysiloxane thermoset material [26]. In most cases, a short aliphatic chain was introduced into the polymer backbone in order to provide flexibility between more rigid aromatic sections, thus improving the processability of the polymer [27,28,29]. For example, Mialon et al. prepared monomers through the reaction of vanillic acid with chloroalkyl alcohols, which provided a tunable aliphatic ‘spacer’ between aromatic units in the resulting polymers with higher thermal stability and satisfactory T_g_ and T_m_ comparable to those of PET [30]. The shortcoming of this study lies with the use of high cost and relatively environmentally unfriendly chloroalkyl alcohols. Chen et al. obtained a series of new bio-based polyesters by the polycondensation of novel monomers with hydroxyl and carboxyl groups prepared by aldol condensation of aromatic aldehydes with levulinic acid [31]. Although significant achievements have obtained during the past five years, some of studies still suffered drawbacks of using complex, multiple synthetic steps and non-sustainable feedstocks to prepare polymeric monomers. Therefore, there are still great interests in new approaches to prepare vanillin-based aromatic monomers and their polymers.

Diols are important for polymer synthesis, and vanillin can be readily converted to vanillyl alcohol, a diol, by the reduction of the aldehyde with sodium borohydride. The phenolic alcohol can also, if desired, be functionalized to introduce an aliphatic alcohol. Reza et al. functionalized this phenolic position on vanillin by reacting it with 2-chloroethanol with catalytic potassium carbonate at reflux in DMSO for 6 h obtained 75% yield of 4-(2-hydroxyethoxy)-3- methoxybenzaldehyde [32]. Yang et al. performed a similar reaction in dimethylformamide (DMF) at 100 °C for 5 h to obtain a yield only of 57% [33]. Yosuke et al. used acetonitrile as solvent at reflux for 48 h to obtain a yield only of 42% [34]. Each of these solvents has problematic aspects from a green chemistry perspective, with DMSO being relatively low-toxicity but having issues with the generation of SO_x_ when incinerated, and both acetonitrile and especially DMF have problematic toxicity issues. Ethylene carbonate, by contrast, is regarded as a non-toxic and “green” solvent or reagent produced by the cyclic addition reaction of carbon dioxide with ethylene oxide [35,36,37]. Ethylene oxide can itself can also be attained from renewable resources [38].

Herein, vanillin was firstly converted into 4-(2-hydroxyethoxy)-3-methoxybenzaldehyde using ethylene carbonate as a hydroxyethylation reagent under solvent-free conditions catalyzed by cheap ionic liquids, and then the hydroxyethylated vanillin was reduced to 2-(4-(hydroxymethyl)-2-methoxyphenoxy) ethanol using green reducing agent sodium borohydride, giving a useful monomer for the synthesis of series of new polyesters (PE) and polyurethanes (PU). For comparison, the direct reduction of vanillin to produce 4-(hydroxymethyl)-2-methoxyphenol and hence its polymers is also discussed. The structure and thermal properties of as-prepared polyesters and polyurethanes were confirmed and evaluated by various characteristics.

## 2. Materials and Methods

### 2.1. Materials

Vanillin, ethylene carbonate (EC), tetrabutylammonium iodide (TBAI), oxalyl chloride (OC), succinyl chloride (SC), terephthaloyl chloride (TC), vanillyl alcohol (VA), 2.5- furandicarboxylic acid, thionylchloride, dimethylformamide (DMF), pyridine, hexamethylenediisocyanate (HDI), isophoronediisocyanate (IPDI), diphenylmethanediisocyanate (MDI) and 1,8-diazabicyclo [5.4.0]undec-7-ene (DBU) were purchased from Aladdin. Sodium borohydride (NaBH_4_) was purchased from Kemel. Tetrahydrofuran (THF) and pyridine were distilled from over calcium hydride before use. Other chemicals were used as received.

### 2.2. Procedure for the Synthesis of HMBD

Vanillin (6.147 g, 40 mmol) was dissolved in ethylene carbonate (7.116 g, 80 mmol) at 80 °C in a 100 mL two-neck round bottom flask. Tetrabutylammonium iodide (2.985 g, 8 mmol) was added and the reaction heated at 110 °C for 12 h. When the reaction was cooled to room temperature, deionized water (20 mL) was added into the mixture and then the mixture was extracted with ethyl acetate (3 × 50 mL). The combined organic layers were dried over anhydrous Na_2_SO_4_ and concentrated in vacuo. The crude product was purified by silica gel column chromatography (eluting with ethyl acetate/petroleum ether = 1:1) to afford 4-(2-hydroxyethoxy)-3-methoxybenzaldehyde (HMBD) with yield of 95%.

### 2.3. Procedure for the Synthesis s of HMEO

Sodium borohydride (1.25 g, 9 mmol) was added portion wise to a solution of HMBD (1.961 g, 10 mmol) in 20 mL of methanol and the resulting mixture was stirred at room temperature for 1 h. The crude product was purified by silica gel column chromatograph (eluting with ethyl acetate/petroleum ether = 1:1) to afford 2-(4-(hydroxymethyl)-2-methoxyphenoxy) ethan-1-ol (HMEO) with a yield of 81%.

### 2.4. Procedure to Prepare PEs

**Scheme 1:**
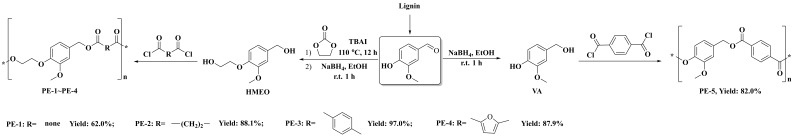
A green and sustainable pathway to prepare bio-based polyesters from vanillin and ethylene carbonate.

The diacyl chloride (2.0 mmol) was dropwise added to a stirred solution of HMEO (0.396 g, 2.0 mmol) and anhydrous pyridine (0.354 mL, 4.4 mmol) in anhydrous THF (10 mL) under nitrogen at 0 °C. The reaction was allowed to warm to room temperature and stirred for 24 h, after which the reaction mixture was precipitated in methanol. The solid polymer was isolated by filtration, and washed three times with cold methanol, and dried at 40 °C under vacuum for 24 h (Scheme 1).

### 2.5. Procedure to Prepare PUs

**Scheme 2:**
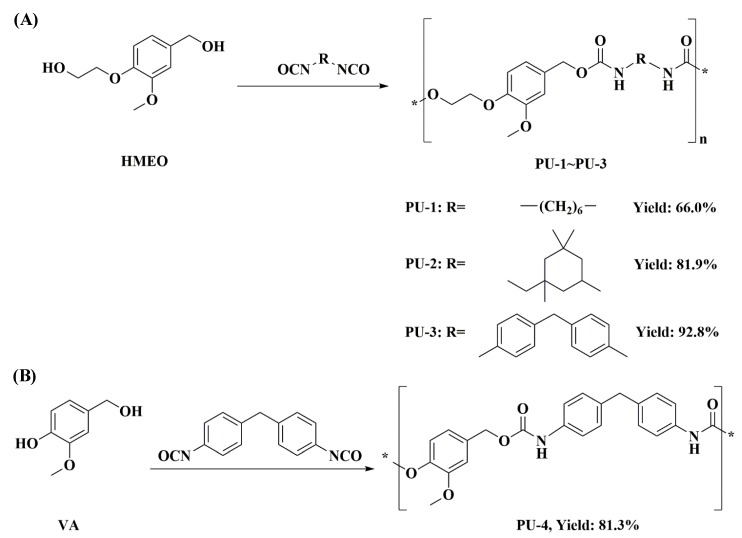
(**A**): The reaction of HMEO with diisocyanatos to prepare PUs; (**B**): the reaction of vanillyl alcohol (VA) with diphenylmethanediisocyanate to prepare polyurethane.

To a solution of HMEO (0.396 g, 2.0 mmol) in anhydrous THF (10 mL) was added diisocyanate (2.0 mmol) and DBU (3 mol%, based on HMEO), the mixture was stirred at 30 °C for 24 h, after which the reaction the mixture was precipitated into methanol. The polymers were obtained after three times washing with methanol and drying at 40 °C under vacuum for 24 h (Scheme 2).

### 2.6. Molecular, Thermal and Structural Characterization

NMR spectra were recorded on a JOEL ECX500 spectrometer referenced to tetramethylsilane (400 MHz for ^1^H NMR and 101 MHz for ^13^C NMR). Fourier-transform infrared (FTIR) spectra were collected on a Thermoscientific Nicolet iS50 instrument at room temperature in the range of 500–4000 cm^−1^. Polymer molecular weight was measured by gel permeation chromatography (GPC) with DMF with 0.01 M LiBr as the eluent, at 40 °C against polystyrene (PS) calibration standards. GPC was performed on a Shimadzu Prominence-i LC-2030C 3D equipped with both a refractive index (RI) and a photo-diode array (PDA) detector, using a Shodex PGC KD-804 column. Thermogravimetric analyses (TGA) were performed on a TA Instruments Q500 instrument at a heating rate of 10 °C/min under nitrogen atmosphere. Differential scanning calorimetry (DSC) analyses were performed on a TA Instruments Q2000 using hermetically-sealed T-zero aluminum pans. The glass transition temperature (T_g_) and melting point were taken from the second heating cycle at a rate of 10 °C/min under nitrogen atmosphere.

## 3. Results and Discussion

### 3.1. Structural Analysis of Monomers

**Figure 1:**
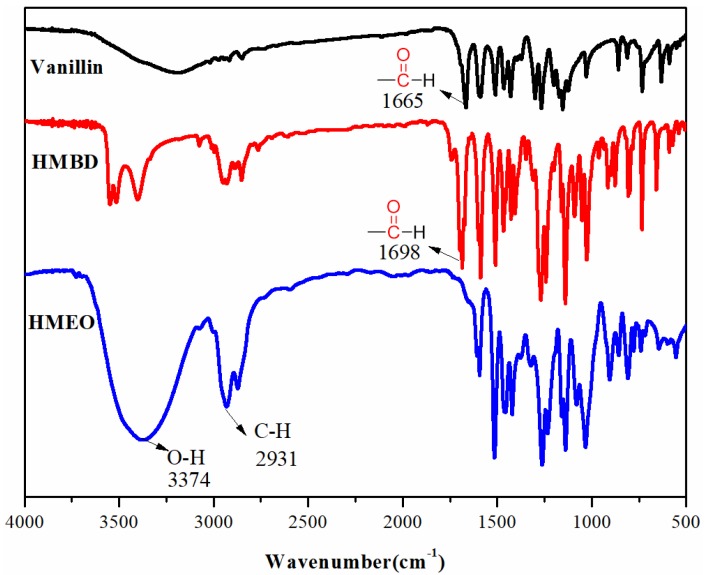
FTIR spectra of vanillin 4-(2-hydroxyethoxy)-3-methoxybenzaldehyde (HMBD) and 2-(4-(hydroxymethyl)-2-methoxyphenoxy) ethan-1-ol (HMEO).

It was found that the vanillin reacted with ethylene carbonate with no additional solvent, catalyzed by cheap and recyclable tetrabutyl ammonium iodide, producing hydroxyethylated vanillin (HMBD) in 95% yield. Subsequently, the formyl group in HMBD was reduced with sodium borohydride giving vanillin-based diol monomer (HMEO) in 81% yield. The formation of HMBD and HMEO were confirmed by ^1^H NMR and ^13^C NMR. In the ^1^H NMR spectrum of HMBD the chemical shift at 4.09 and 3.75 ppm are assigned to the newly formed methylene groups from ethylene carbonate (Figure S1). Meanwhile in the ^13^C NMR spectrum the chemical shift of newly formed methylene appeared at 70.4 and 59.2 ppm (Figure S2). After reduction, the formyl group transformed to hydroxyl group which was demonstrated by the disappearance of the peak at 9.83 ppm and the appearance of 5.08 and 4.42 ppm in the ^1^H NMR spectrum of HMEO (Figure S3). In addition, in the ^13^C NMR spectrum (Figure S4), the complete disappearance of 191.4 ppm and the formation of 62.9 ppm indicated the successful reduction. The obtained HMBD and HMEO were also characterized by FTIR (Figure 1). Compared with vanillin the HMBD shown new peak at 2931 cm^−1^ which can be attributed to the stretching vibration of newly formed methylene. After reduction, the sharp peak at around 1690 cm^−1^ assigned to stretching vibration of the carbonyl vanished completely. Meanwhile the strong peak at around 3374 cm^−1^ can be assigned to the stretching vibration of the hydroxyl in HMEO. The results of NMR and FTIR illustrated the successful synthesis of vanillin-based diol monomer.

### 3.2. Structural Analysis of PEs

With the vanillin-based diol monomer in hand, we prepared polyesters and polyurethanes with diacyl chlorides and diisocyanatos, respectively according to the literature methods [20,39]. A series of polyesters were obtained via direct acylation between HMEO and acyl chlorides including oxalyl chloride (OC), succinyl chloride (SC) [40], terephthaloyl chloride (TC) [41] and 2,5-furandicarbonyl dichloride (FC) [42,43]. The 2,5-furandicarboxylic acid has been extensively studied in bio-based polymers [44,45,46]. For example, Mou et al. prepared a series of furan-based polyester prepared using furyl diol, and found that these polyesters had good thermal properties [47]. Genovese et al. prepared a furan-based polyester through a solvent-free process, starting directly from 2,5-furandicarboxylic acid, the polyester was shown to have excellent oxygen barrier performance superior to PEF [48]. Therefore, we decided to introduce the furan ring into the vanillin-based polyester, in the hope of obtaining bio-based polyesters benefiting from similarly enhanced properties. The 2,5-furandicarbonyl dichloride was prepared according to the literature method [49], and used immediately in the polymerization reaction.

The polymerization was carried out in THF at room temperature for 24 h using pyridine as a catalyst. After precipitation, all of the polyesters were obtained in high yields (82–97%) except PE-1 (62%). Of the polyesters derived from TC, PE-3 show higher yield than PE-5, which may be attributed to the lower reactivity of the phenolic hydroxyl as compared to the aliphatic alcohol.

**Figure 2:**
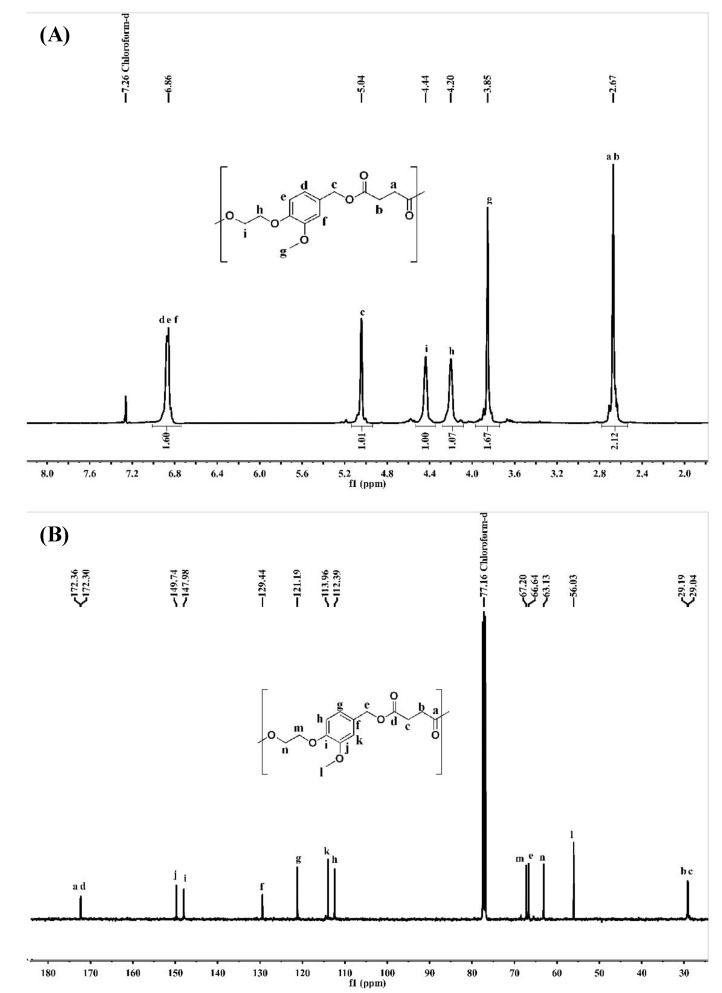
^1^H (**A**) and ^13^C NMR (**B**) spectra of PE-2.

**Figure 3:**
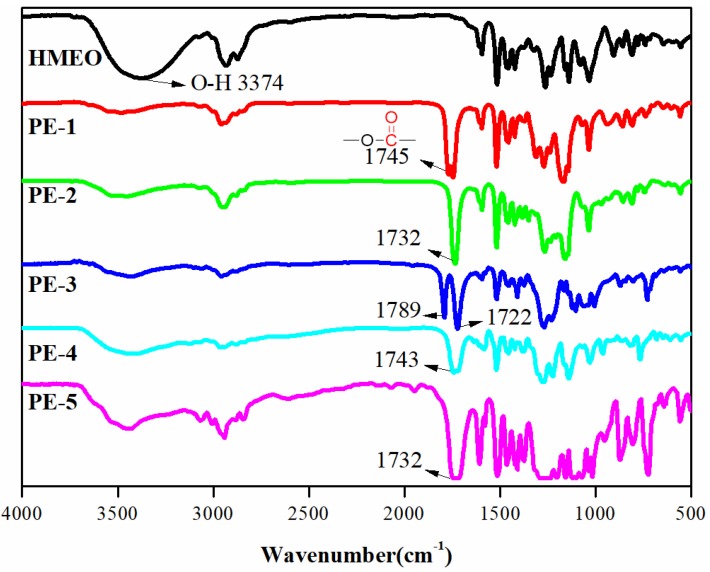
FTIR spectra of PEs and HMEO.

The structures of obtained polyesters were characterized by NMR and FTIR. Taking PE-2 as a represented example, the chemical shift of hydroxyl at 5.08 and 4.84 ppm disappeared completely while the chemical shift belonging to newly formed methylene appeared at 2.67 ppm in the ^1^H NMR spectrum (Figure 2A). This result was affirmed by the ^13^C NMR spectrum (Figure 2B), in which the newly formed methylene appeared at 29.0 ppm while the -C=O appeared at 172.4 ppm. In the FTIR the newly formed strong peak at around 1740 cm^−1^ is due to the stretching vibration of -C=O. (Figure 3).

### 3.3. Molecular, Thermal and XRD Analysis of PEs

The GPC and NMR results demonstrated the formation of polyesters with high molecular weight. The M_n_ of obtained polyesters were in the range of 17 to 36 KDa with PDI ranging from 1.05–1.14. The M_n_ increased as the hindrance of acyl chloride decreased and the use of SC resulted in the highest M_n_ of 36 KDa (Table 1).

**Figure 4:**
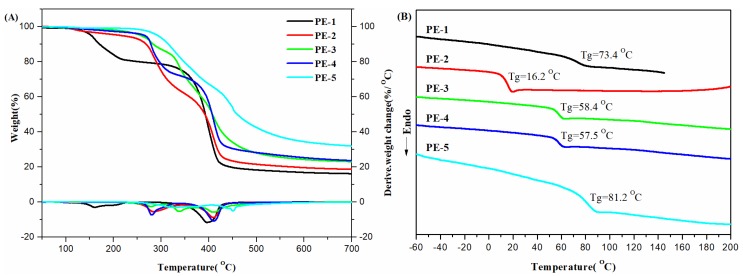
TGA curves (**A**) and differential scanning calorimetry (DSC) traces (**B**) of PEs.

**Figure 5:**
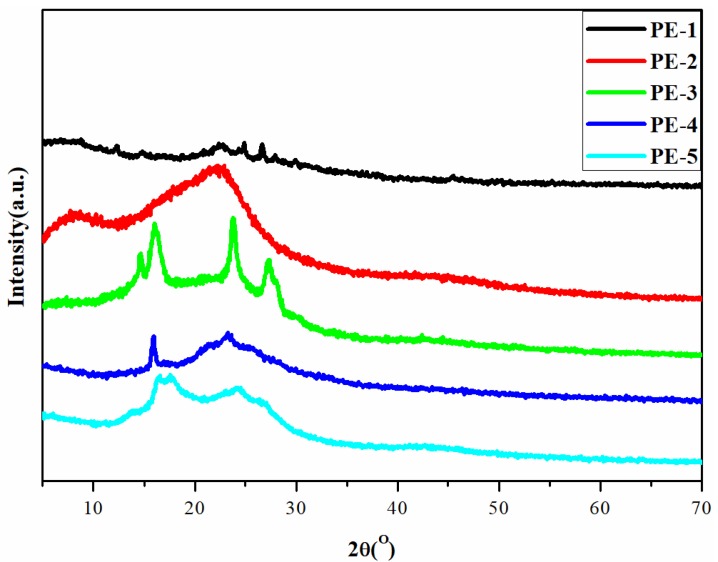
XRD curves of PEs.

The thermal properties of the PEs were examined by TGA (Figure 4A, Table 1) and DSC (Figure 4B, Table 1). PE-2, PE-3 and PE-4 showed higher thermal stability with 5% mass loss occurring at 214, 257 and 264 °C, respectively, and the thermal properties of PE-3 and PE-4 are very similar. While PE-1 exhibited lower thermal stability with 5% mass loss occurred at 153 °C. Compared with PE-5, due to the presence of short fatty segments in PE-3, the thermal stability with 5% mass loss occurring at 257 °C, PE-5 showed higher thermal stability with 5% mass loss increased to 293 °C. All polyesters underwent at least two stages of decomposition, likely due to the breakdown of ester bonds and benzene rings. The residual weight at 700 °C decreased as follow: PE-5 >PE-4 >PE-3 > PE-2 >PE-1, implying that residual weight is related to the rigid aromatic group content for the polyester. The T_g_ increased from 16.2 to 73.4 °C as the carbon number of acyl chloride decreased from 4 to 2, which may due to the decreased flexibility of the aliphatic chain (Table 1, PE1 and 2). While using TC or FC, polyesters with high T_g_ were obtained (Table 1, PE3 and 4), the T_g_ of PE-4 is similar to PE-3 indicating that the furan-based polyester has similar properties to the benzene-based polyester, and their T_g_s are much higher than that of PBT (38 °C). Compared with PE-3, PE-5 showed increased T_g_ as the rigidity of the diol increased and both their T_g_s are comparable or even higher than those of PBT and PET (38 and 67 °C, respectively) [31]. Although no melting point was observed for any of these polyesters, the XRD results indicated that the PE-2 is amorphous, whilst the other polyesters contain partial crystalline regions (Figure 5).

### 3.4. Structural Analysis of PUs

Polyurethane has been widely used in various applications including upholstery, building materials, and transportation [50,51]. The traditional method to prepare polyurethanes is via the polyaddition of polyols and polyisocyanates [52]. Due to the high atom economy of this polyaddition, the use of bio-based polyols is particularly interesting to replace the use of less sustainable petroleum based monomers in these applications [53,54]. Therefore, a series of vanillin-based polyurethanes were prepared by polyaddition of HMEO monomer and diisocyanatos catalyzed by DBU in THF, including hexamethylenediisocyanate (HDI), isophoronediisocyanate (IPDI), diphenylmethanediisocyanate (MDI). In contrast, PU-4 was prepared from vanillyl alcohol (VA) and diphenylmethanediisocyanate (MDI). It is worth mentioning that isocyanates do have significant issues with their toxicity and hazardous, vigorous reactivity with moisture, and we acknowledge that much important work is being done (including by these authors) on non-isocyanate polyurethanes (NIPUs) [55,56]. However, these technologies, whilst safer, are not yet mature. The development of bio-based polyols offers a more sustainable alternative (compared conventional methods) that might realistically be brought into use in a far shorter timescale, indeed soybean-derived polyols have been used commercially in car seat foams for many years [57].

**Figure 6:**
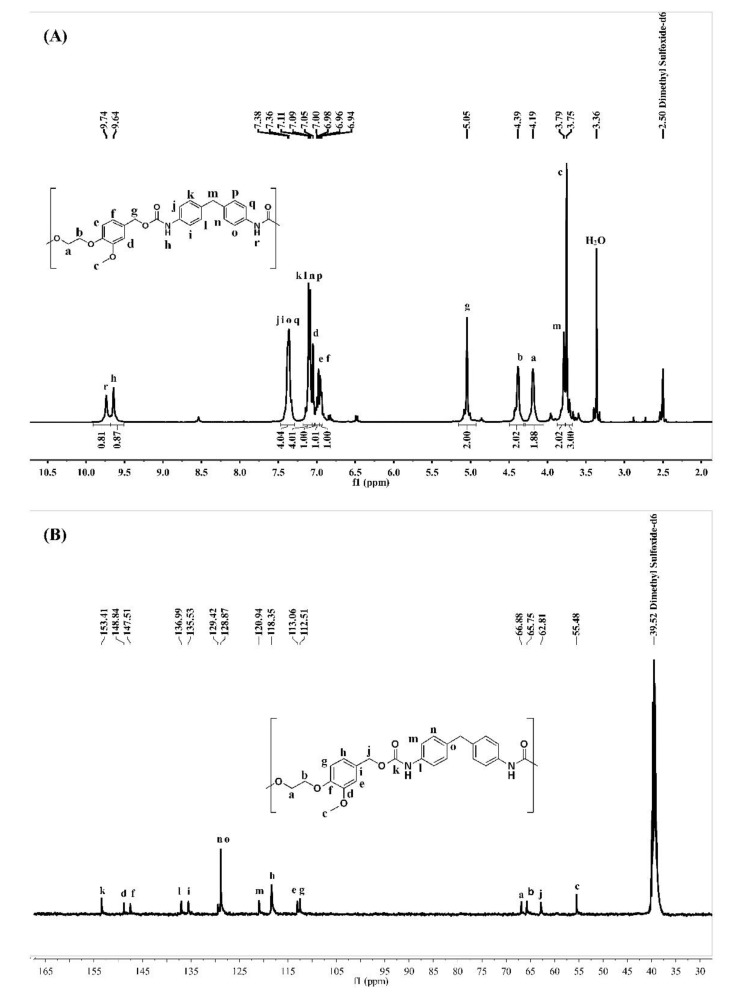
Comparative ^1^H NMR spectra of PU-3 (**A**) and ^13^C NMR spectra of PU-3 (**B**).

The structures of PUs were determined by ^1^H NMR and ^13^C NMR (Figures S15–S22). Taking PU-3 as an example, there are no peaks of hydroxyl group at 5.08 ppm and 4.84 ppm, while the typical chemical shifts of amide proton appears at 9.64 ppm and 9.74 ppm. The multiple peaks at 7.38–7.05 ppm represent the characteristic peaks of benzene ring (Figure 6A). In the ^13^C NMR spectrum of PU-3 (Figure 6B) the chemical shift at 153.4 ppm can be assigned to the new carbonyl environment of the urethane functionality.

**Figure 7:**
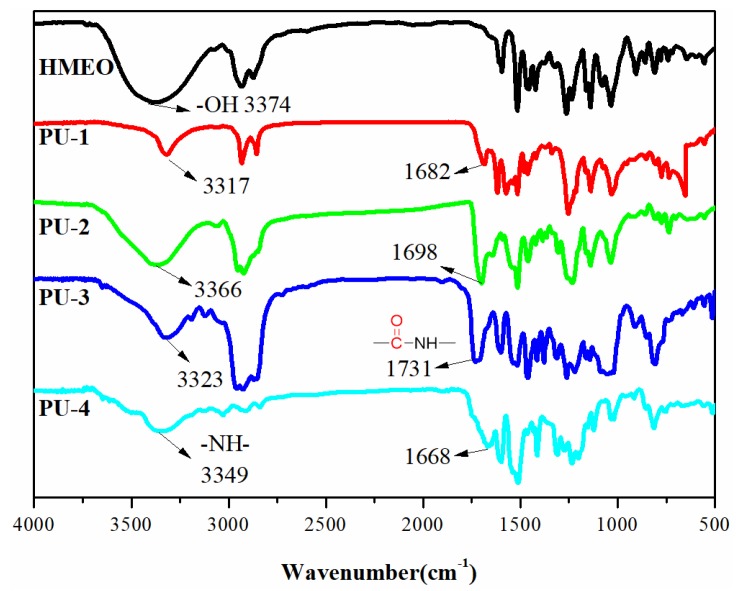
FTIR spectra of PUs and HMEO.

The formation of these PUs were further confirmed by FTIR (Figure 7). In the FTIR spectra of PUs the hydroxyl peak at 3374 cm^−1^ is replaced by the amino at 3317 cm^−1^. The new strong signal at 1698 cm^−1^ further demonstrates the presence of the urethane backbone.

### 3.5. Molecular, Thermal and XRD analysis of PUs

**Figure 8:**
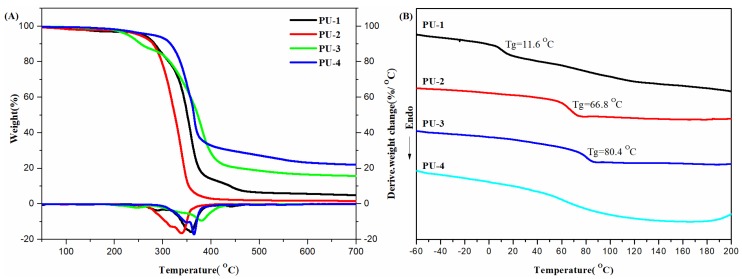
TGA curves (**A**) and DSC traces (**B**) of PUs.

The yields of those PUs obtained from HMEO are above 80%, except PU-1 which give a more modest yield of 66% (Table 2). PU-4 derived from vanillic alcohol exhibits a lower yield of 81.3% as compared with PU-3, which may due to the lower activity of phenolic hydroxyl. The GPC results reveal that PUs of high Mn are successfully prepared, with M_n_ and PDI in the range of 21–40 KDa and 1.10–1.23, respectively. Compared with PU-3, PU-4 show lower M_n_, which may be attributed to the lower reactivity of the phenolic hydroxyl as compared to the aliphatic alcohol. The thermal properties of the PUs are examined by TGA (Figure 8A, Table 2) and DSC (Figure 8B, Table 2). TGA reveals that all of these polyurethanes have excellent thermal stability, with an initial decomposition temperature (5% weight loss) between 229–280 °C. The initial decomposition temperature of PU-2 is higher than that of PU-3, indicating that the thermal stability of this IPDI-based polyurethane is higher than the aromatic-based polyurethane. Due to the presence of flexible aliphatic segments in PU-3% mass loss occurred at 229 °C, whereas PU-4 with a more rigid vanillic diol derived backbone shows higher thermal stability with 5% mass loss increases to 280 °C and no observed T_g._

**Figure 9:**
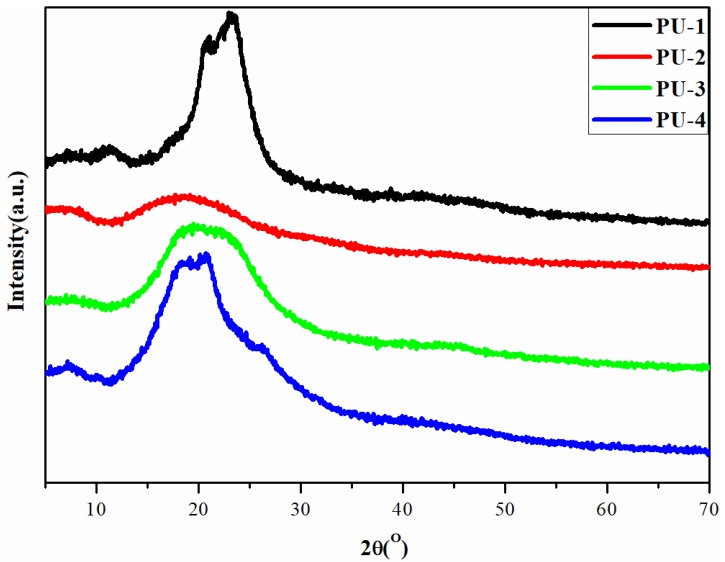
XRD curves of PUs.

The T_g_ of PUs increases from 11.6 to 80.4 °C with increased chain rigidity. The T_g_ of IPDI- based PU-2 and MDI-based PU-3 is 66.8 °C and 80.4 °C, respectively (Table 2, Run 2 and 3) and this is likely because the MDI-based polyurethane backbone has higher rigidity than the aliphatic-isocyanate IPDI-based polyurethane [58]. The initial decomposition temperature of PU-3 is lower than PU-4 due to the presence of two methylene soft segments on the main chain of PU-3. PU-4 shows no T_g_ before the initial decomposition temperature. No melting point was detected for any of these PUs, indicating that they were amorphous, which was also consistent with the XRD data (Figure 9).

## 4. Conclusions

In this paper, HMBD and HMEO have been synthesized in high yields starting from bio-based vanillin and ethylene carbonate via an environmentally sustainable catalytic method. HMEO was employed for the preparation of polyesters and polyurethanes. The results of ^1^H NMR, ^13^C NMR, FTIR and GPC confirmed the successful preparation of high M_n_ polyesters and polyurethanes demonstrating their potential as sustainable polymer materials. The DSC analyses revealed that the T_g_ of obtained polymers were closely correlated with the degree of flexibility (or rigidity) present in the polymer chain. Through PE-3 with PE-5 and PU-3 with PU-4 performance comparison showed that the higher reactivity of the aliphatic hydroxyl group and the flexibility of the alkyl segment had a great influence on the yield, molecular weight and thermal properties of polymer, which provided an important reference for the design of new materials. These two families of lignin-derivable polymers have bio-based content between 44% and 100%, and are therefore of extreme interest as more sustainable future materials.

## Figures and Tables

**Table 1:** Characterization of polyesters prepared from HMBO and vanillyl alcohol (VA) with a range of acyl chlorides.

Sample	Monomer	Acyl Chloride	Yield (%)	M_n_ (g mol^−1^)	PDI (M_w_/M_n_)	T_g_ (DSC) (°C)	T_d_ (5%) (°C)	T_d_ (50%) (°C)
**PE-1**	HMEO	OC	62.0	22,000	1.06	73.4	153	393
**PE-2**	HMEO	SC	88.1	36,000	1.14	16.2	214	392
**PE-3**	HMEO	TC	97.0	17,000	1.05	58.4	257	408
**PE-4**	HMEO	FC	87.9	19,000	1.10	57.5	264	407
**PE-5**	VA	TC	82.0	21,000	1.11	81.2	293	460

**Table 2:** Test data based on the PUs.

Sample	Monomer	Diisocyanate	Yield (%)	M_n_ (g mol^−1^)	PDI (M_w_/M_n_)	T_g_ (DSC) (°C)	T_d_ (5%) (°C)	T_d_ (50%) (°C)
**PU-1**	HMEO	Hexamethylenediisocyanate (HDI)	66.0	39,000	1.23	11.6	259	351
**PU-2**	HMEO	Isophoronediisocyanate (IPDI)	81.9	29,000	1.11	66.8	252	325
**PU-3**	HMEO	Diphenylmethanediisocyanate (MDI)	92.8	40,000	1.11	80.4	229	373
**PU-4**	VA	MDI	81.3	21,000	1.10	-	280	364

## Supplementary Materials

The following are available online at https://www.mdpi.com/2073-4360/12/3/586/s1, Figure S1. ^1^H NMR spectrum of HMBD; Figure S2. ^13^C NMR spectrum of HMBD; Figure S3. ^1^H NMR spectrum of HMEO; Figure S4. ^13^C NMR spectrum of HMEO; Figure S5. ^1^H NMR spectrum of PE-1; Figure S6. ^13^C NMR spectrum of PE-1; Figure S7. ^1^H NMR spectrum of PE-2; Figure S8. ^13^C NMR spectrum of PE-2; Figure S9. ^1^H NMR spectrum of PE-3; Figure S10. ^13^C NMR spectrum of PE-3; Figure S11. ^1^H NMR spectrum of PE-4; Figure S12. ^13^C NMR spectrum of PE-4; Figure S13. ^1^H NMR spectrum of PE-5; Figure S14. ^13^C NMR spectrum of PE-5; Figure S15. ^1^H NMR spectrum of PU-1; Figure S16. ^13^C NMR spectrum of PU-1; Figure S17. ^1^H NMR spectrum of PU-2; Figure S18. ^13^C NMR spectrum of PU-2; Figure S19. ^1^H NMR spectrum of PU-3; Figure S20. ^13^C NMR spectrum of PU-3; Figure S21. ^1^H NMR spectrum of PU-4; Figure S22. ^13^C NMR spectrum of PU-4, which provide ^1^H and ^13^C NMR spectra of monomers and polymers.
