# Supplementary material for: Sustainable Aromatic Aliphatic Polyesters and Polyurethanes Prepared from Vanillin-Derived Diols via Green Catalysis
Source: Polymers (Basel). 2020 Mar 5;12(3):586. doi: 10.3390/polym12030586 (PMC7182816; doi:10.3390/polym12030586)
Supplement: Supplementary file 1 [file polymers-12-00586-s001.pdf]

Supplementary Materials

Sustainable aromatic aliphatic polyesters and polyurethanes prepared from vanillin-derived diols via green catalysis

Changbo Zhao[1], Caijuan Huang[1]*, Qin Chen[1], Ian D. V. Ingram[2], Xiankui Zeng[1], Tianhua Ren[1], Haibo Xie[1]*

[1] Department of Polymer Materials &Engineering, College of Materials & Metallurgy, Guizhou University, West Campus, Huaxi District, Guiyang, China; zcb565782908@163.com (C.Z.);460831749@qq.com(C.H); qchen6@gzu.edu.cn (Q.C.); I.Ingram@mmu.ac.uk (I.I.) ; xk_kkkkk@163.com (X.Z.); thren96@outlook.com (T.R.).

[2] Department of Natural Sciences, Manchester Metropolitan University, Chester Street, Manchester, M1 5DG

* Correspondence: cjhuang98@163.com(C.H.)； hbxie@gzu.edu.cn; Tel.: +86-851-83627806 (H.X.)

SFigures

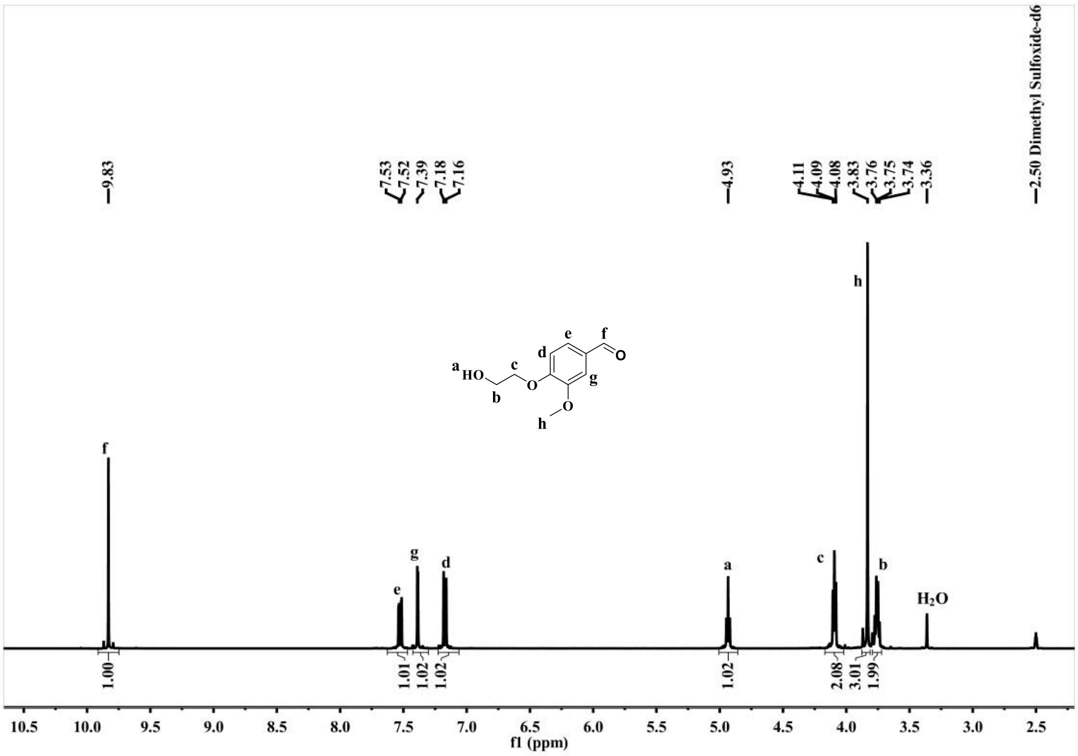

Figure S 1. ^{1}H NMR spectrum of HMBD.

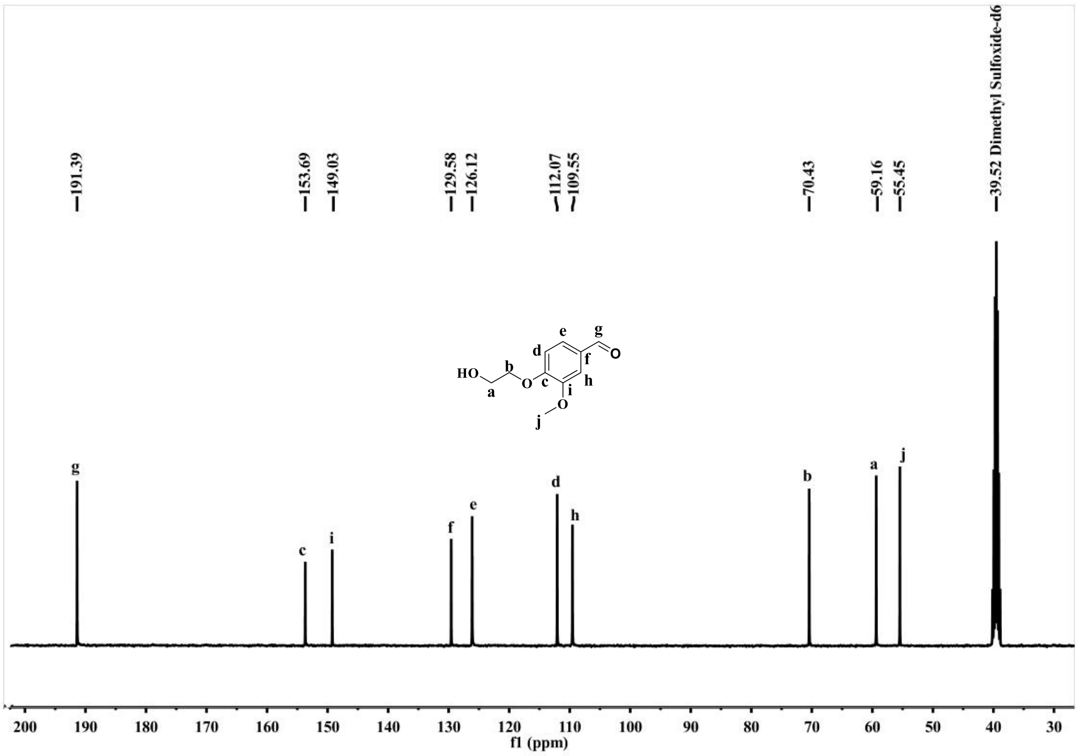

Figure S 2. ^{13}C NMR spectrum of HMBD.

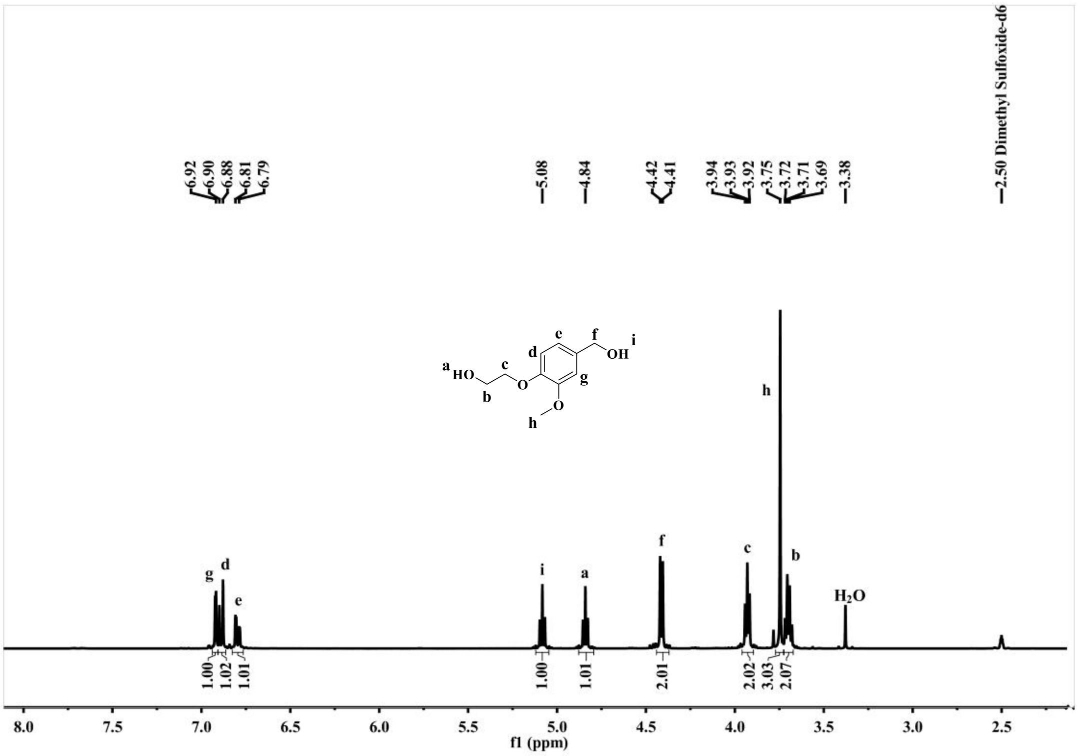

Figure S 3. ^{1}H NMR spectrum of HMEO.

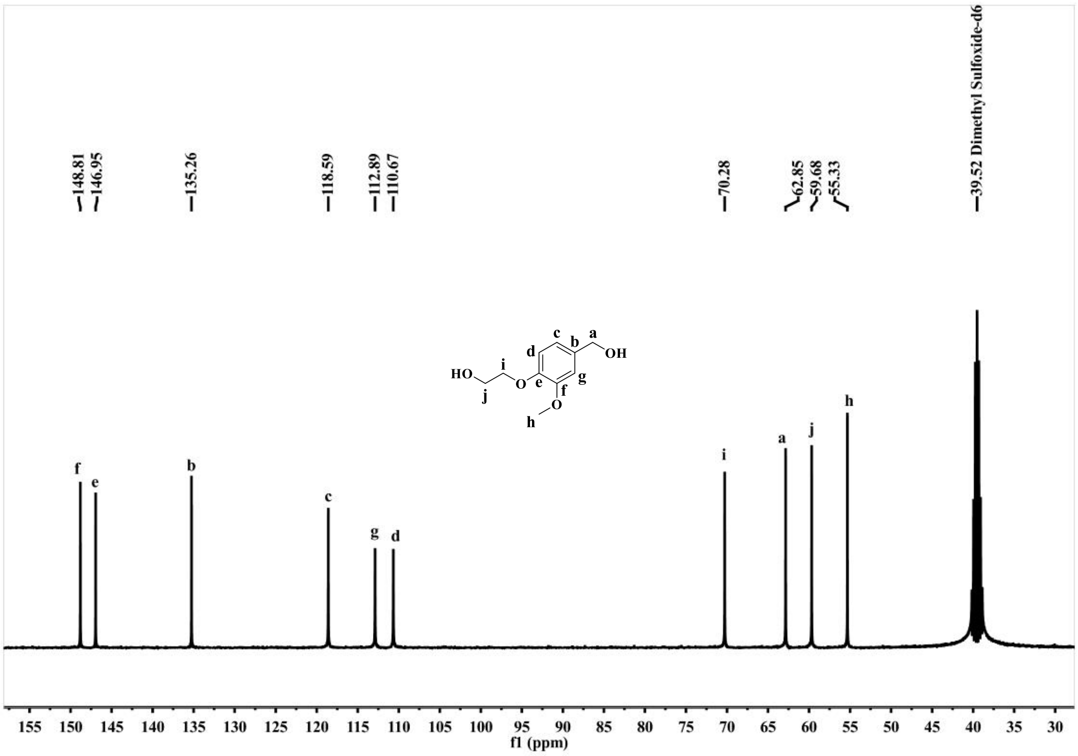

Figure S 4. ^{13}C NMR spectrum of HMEO.

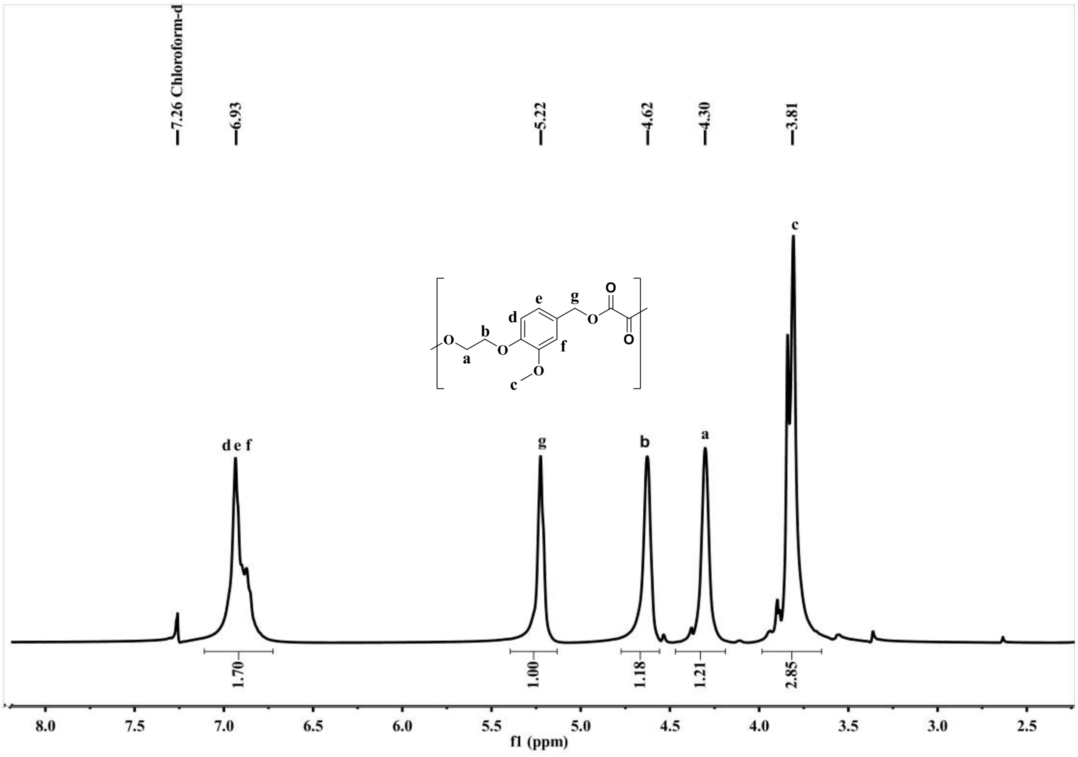

Figure S 5. ^{1}H NMR spectrum of PE-1.

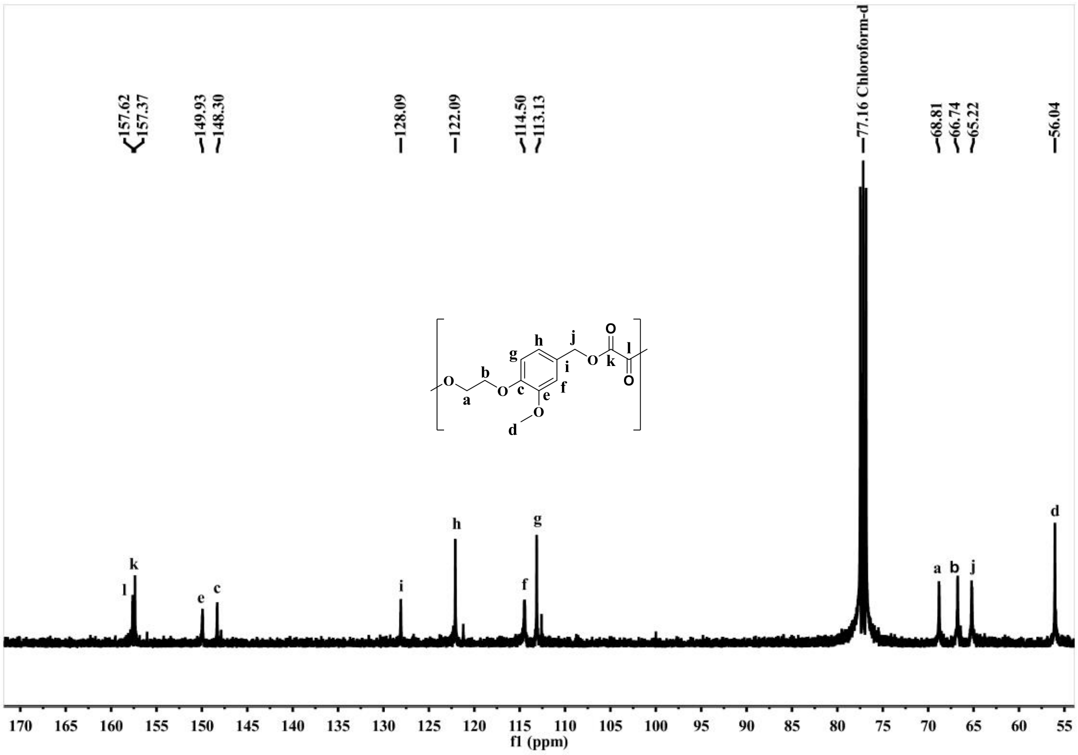

Figure S 6. ^{13}C NMR spectrum of PE-1.

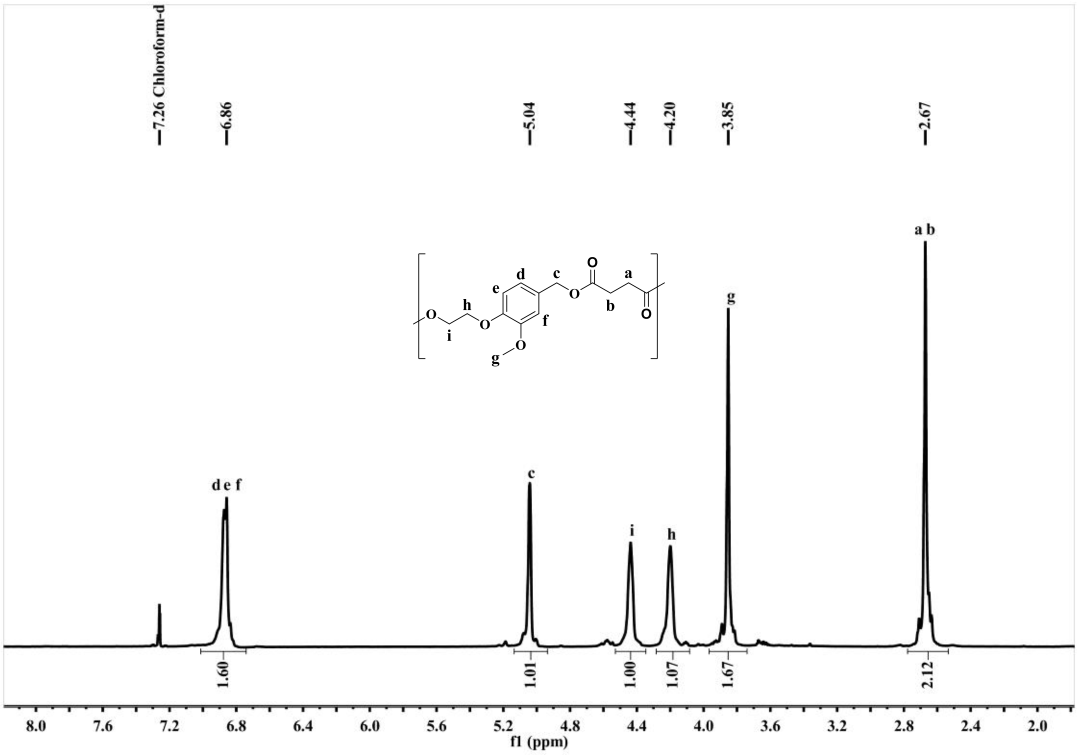

Figure S 7. ^{1}H NMR spectrum of PE-2.

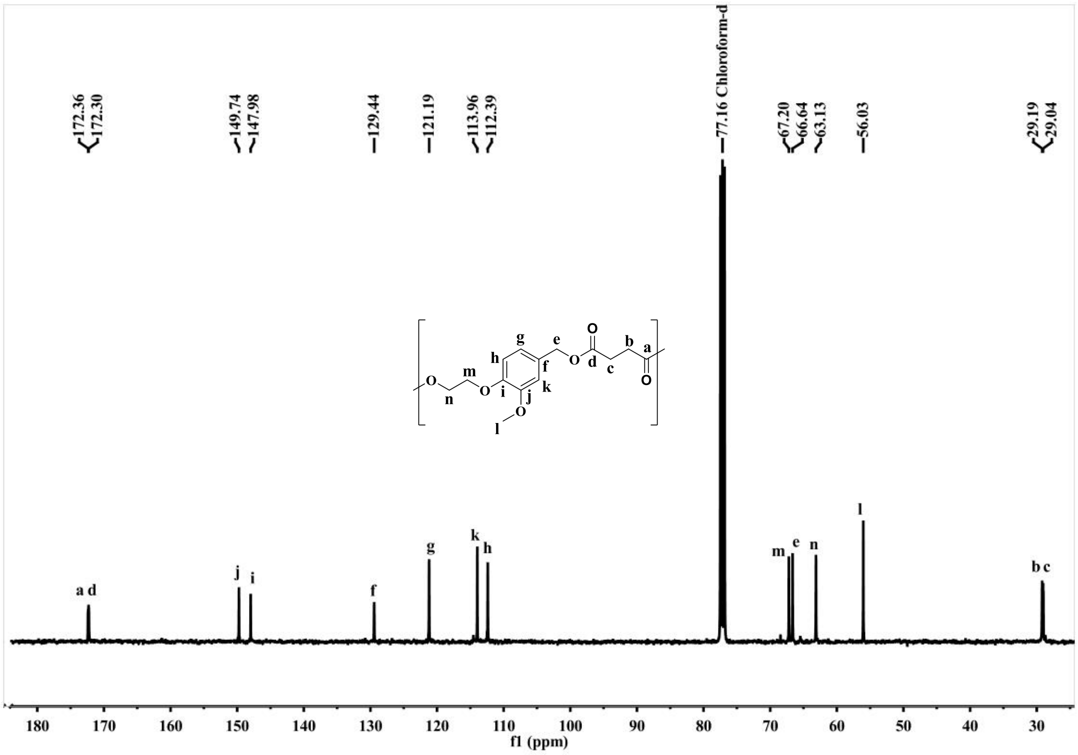

Figure S 8. ^{13}C NMR spectrum of PE-2.

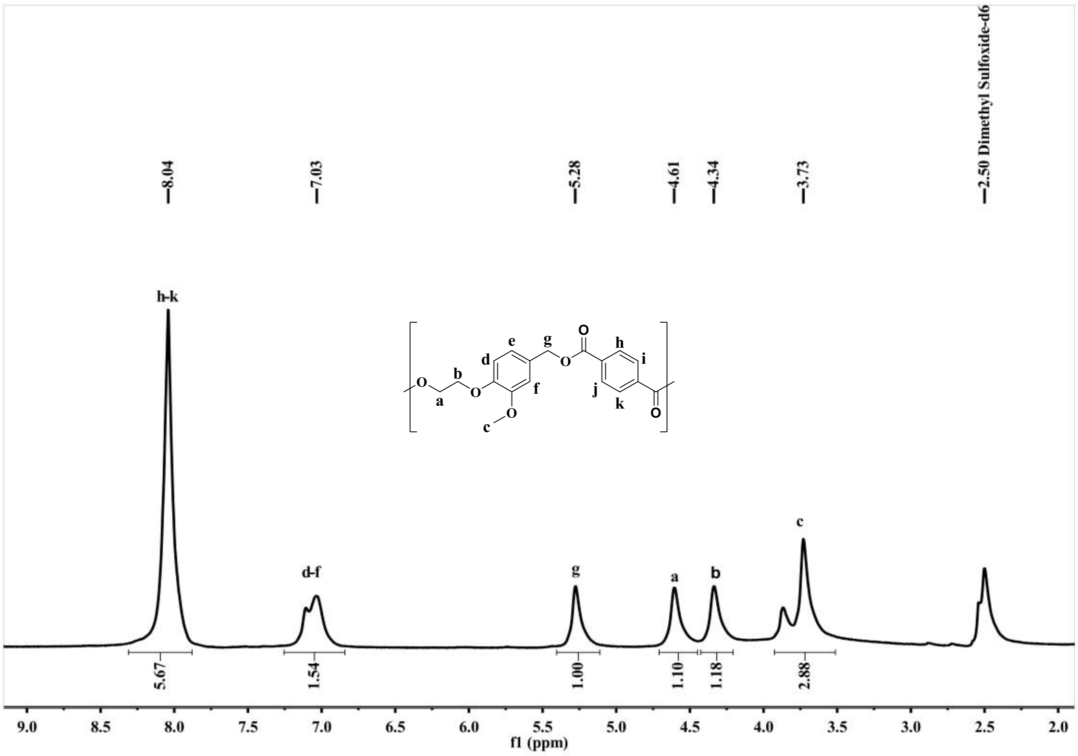

Figure S 9. ^{1}H NMR spectrum of PE-3.

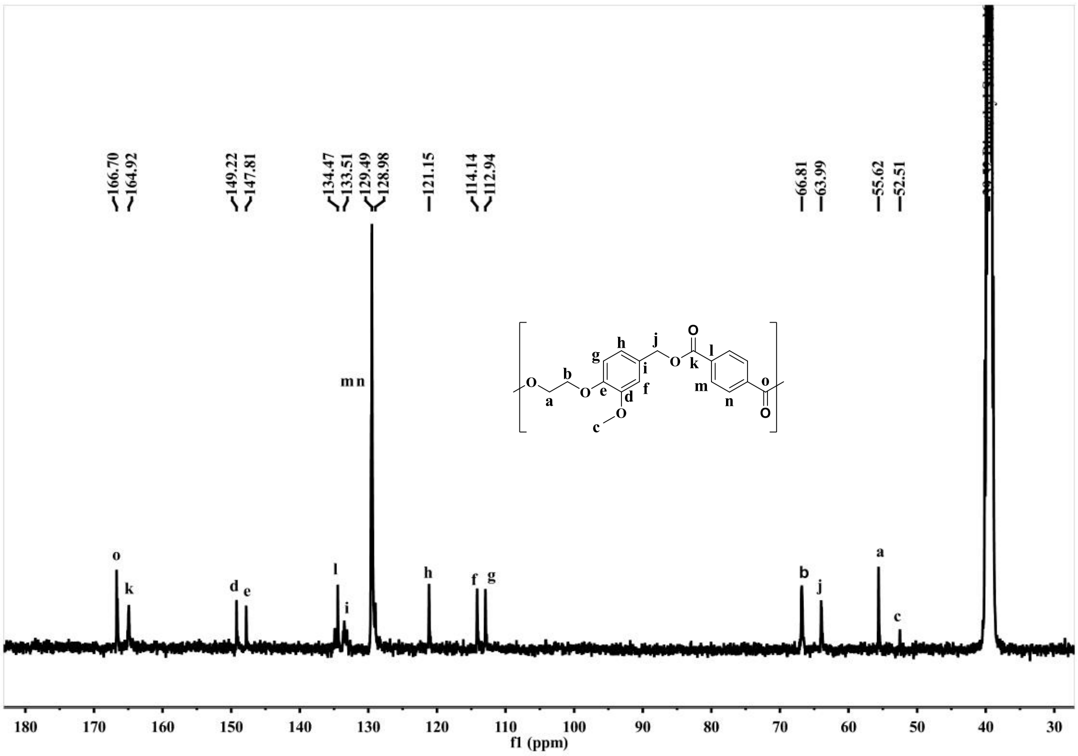

Figure S 10.^{13}C NMR spectrum of PE-3.

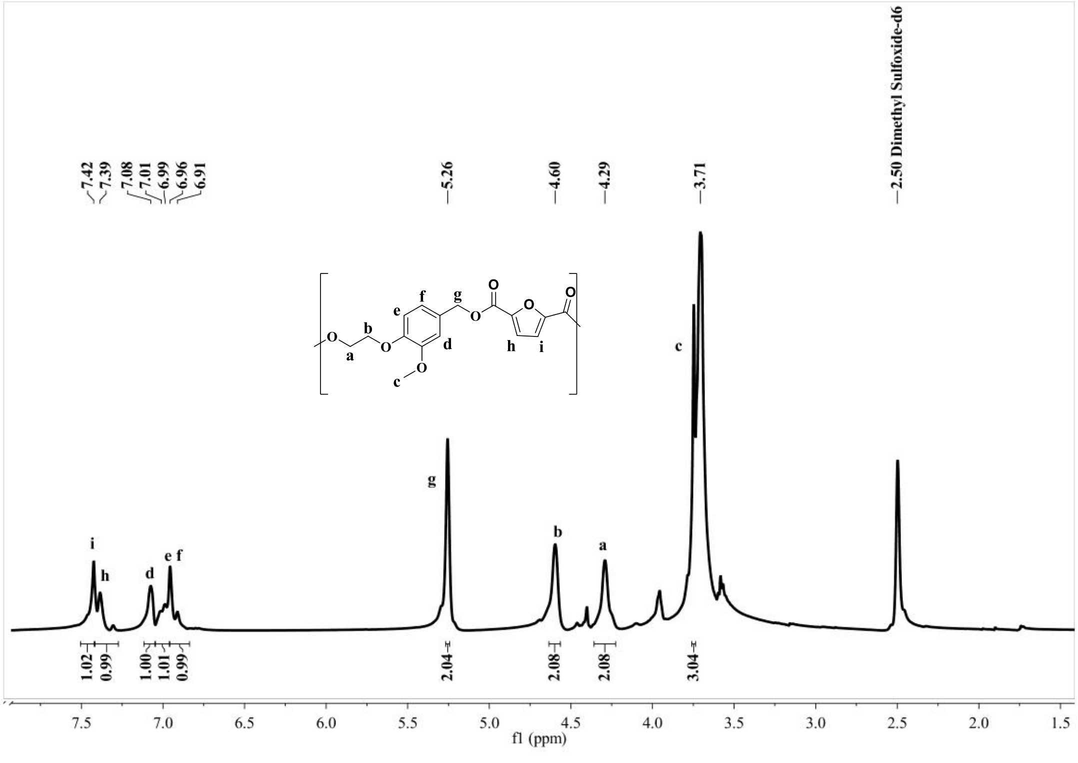

Figure S 11. ^{1}H NMR spectrum of PE-4.

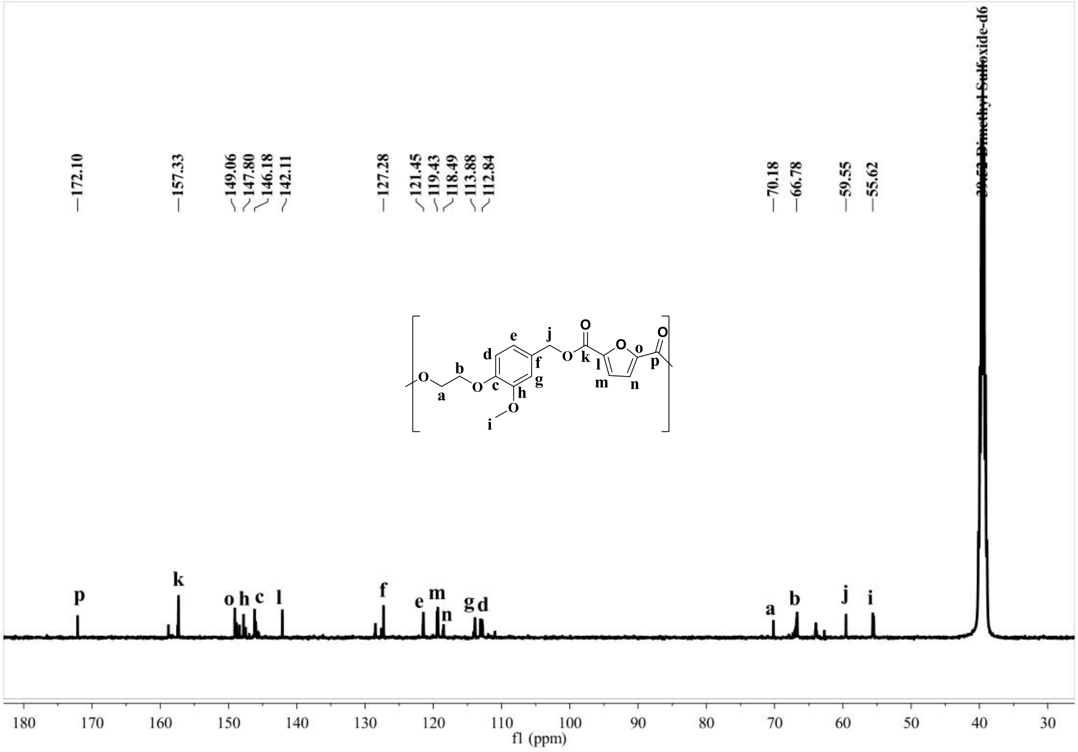

Figure S 12. ^{13}C NMR spectrum of PE-4.

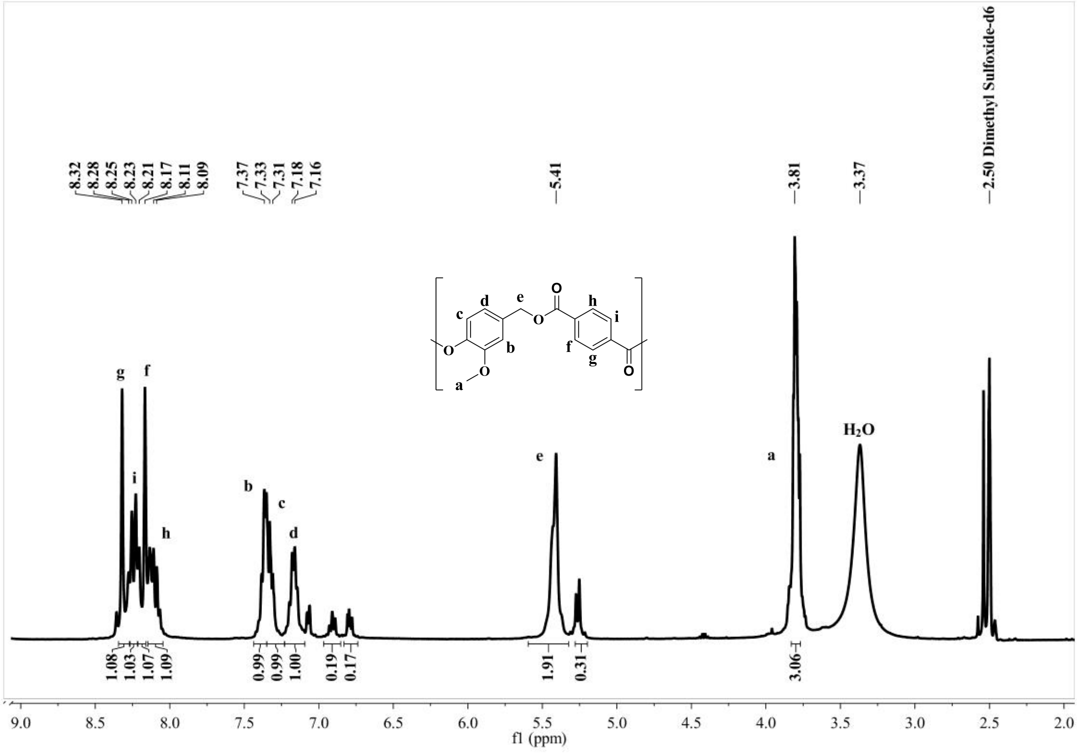

Figure S 13. ^{1}H NMR spectrum of PE-5.

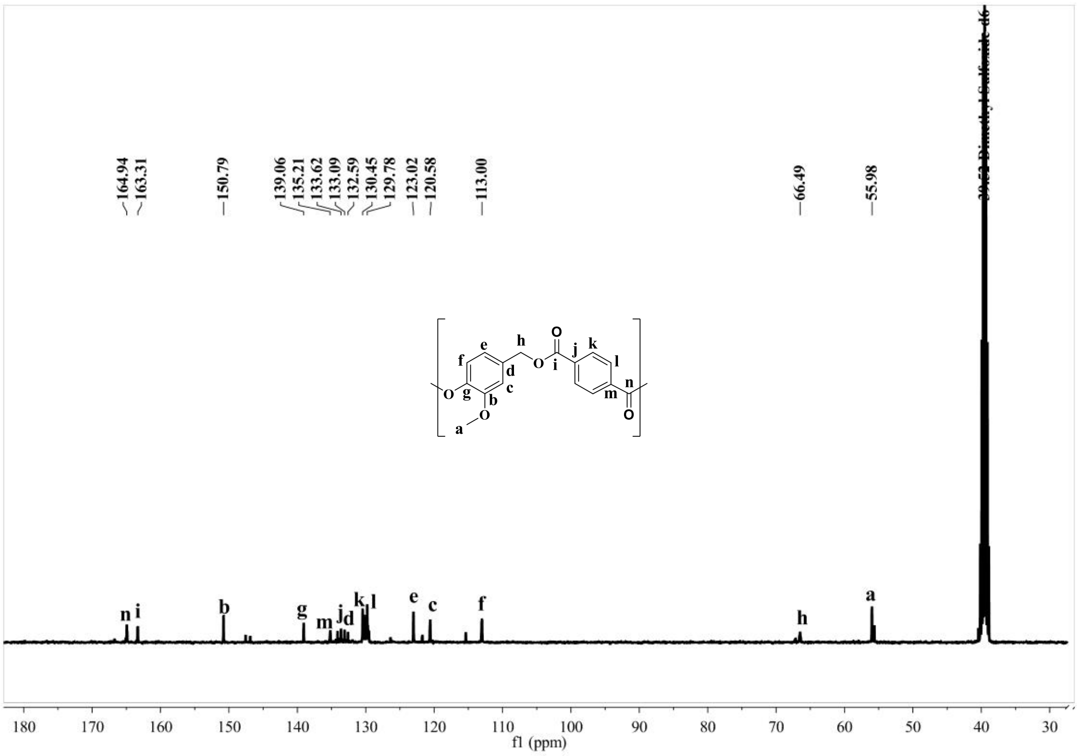

Figure S 14. ^{13}C NMR spectrum of PE-5.

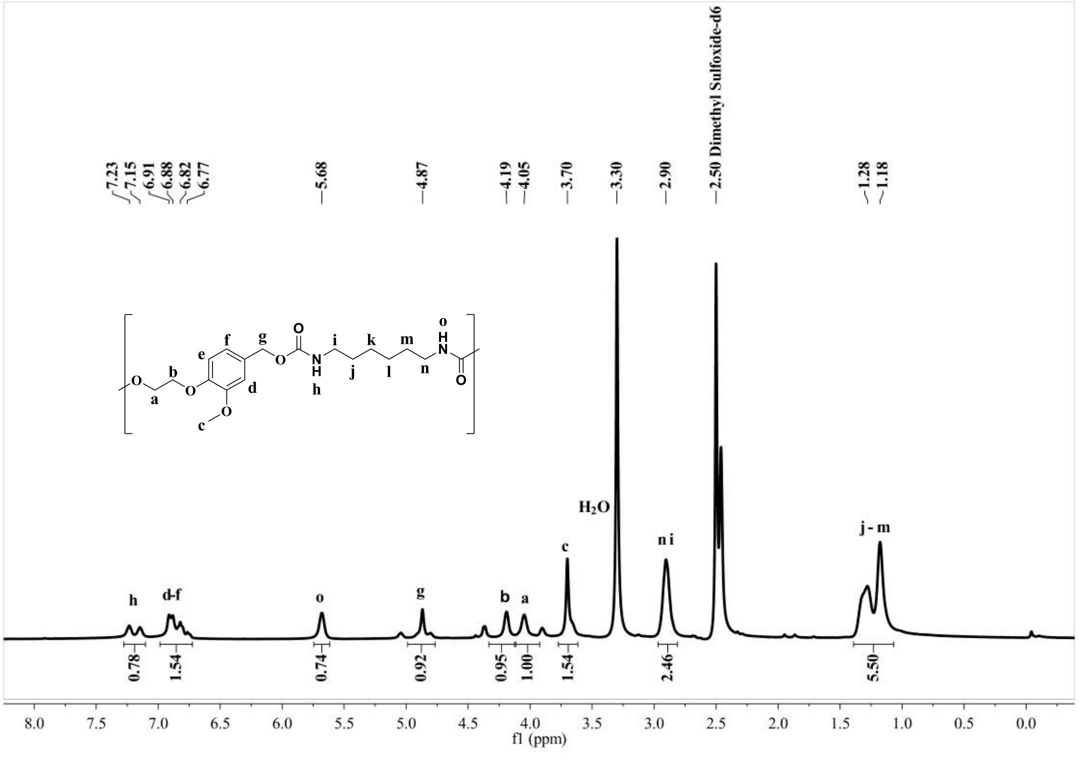

Figure S 15. ^{1}H NMR spectrum of PU-1.

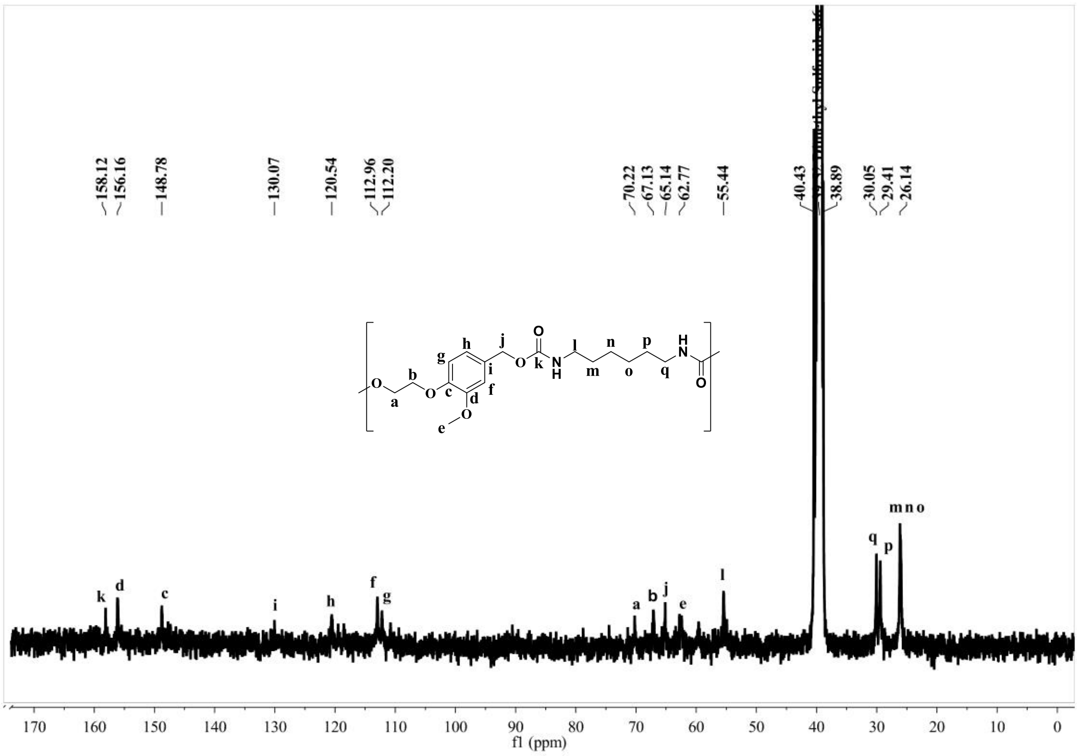

Figure S 16. ^{13}C NMR spectrum of PU-1.

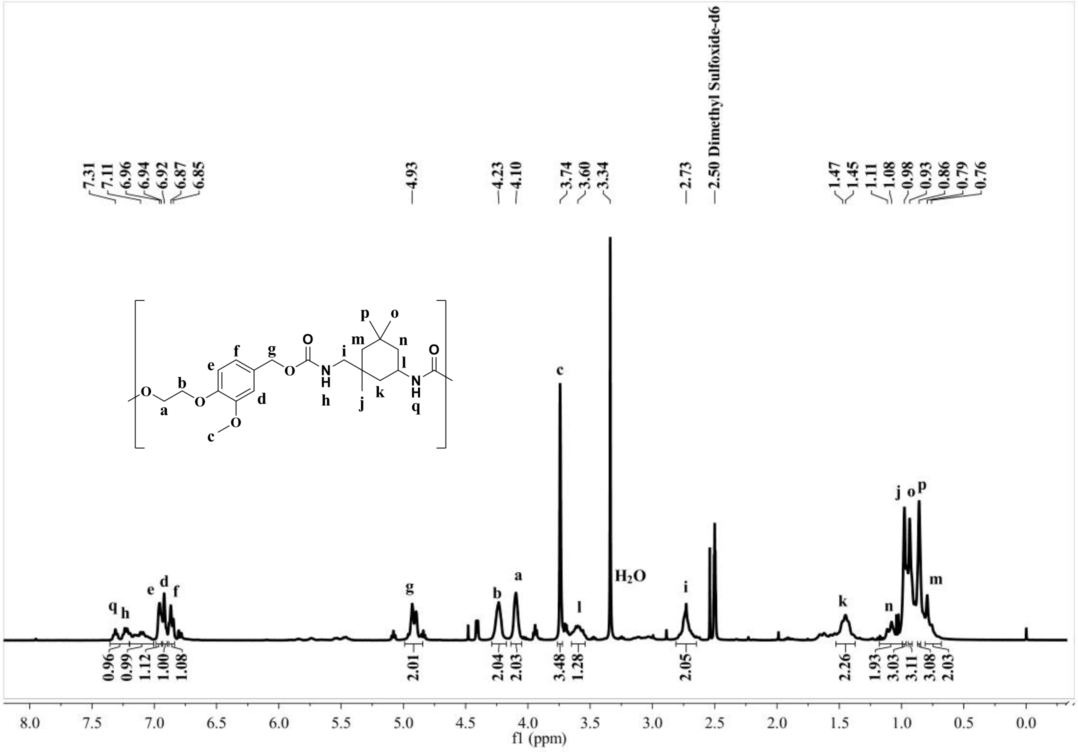

Figure S 17. ^{1}H NMR spectrum of PU-2.

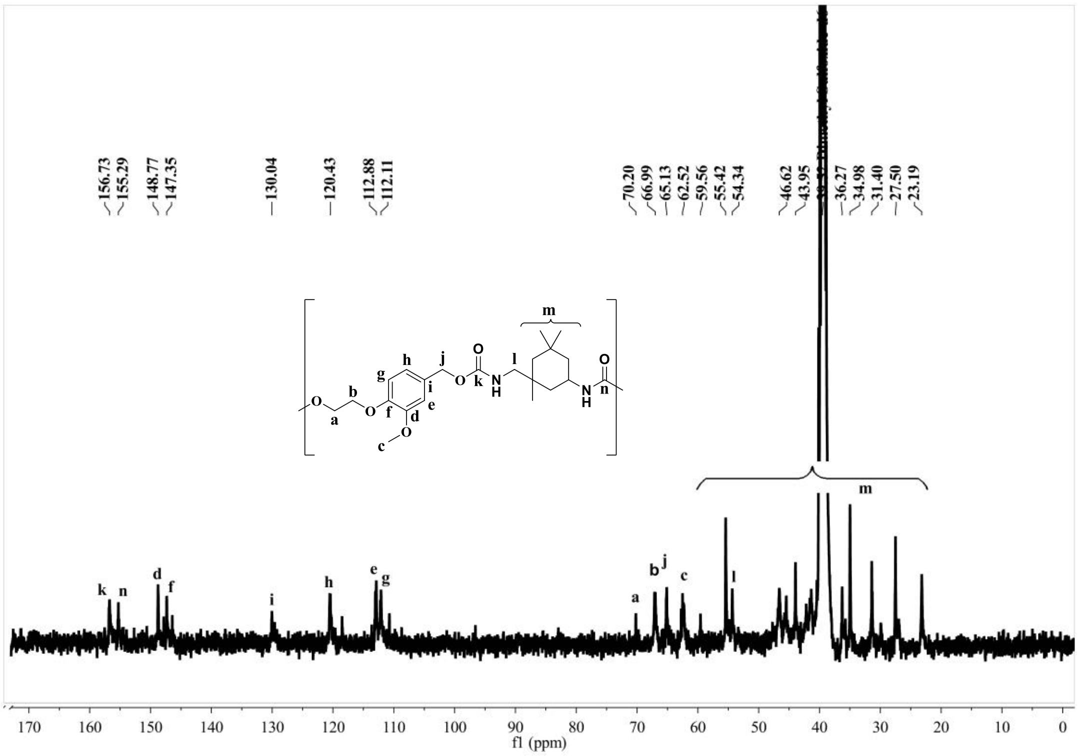

Figure S 18. ^{13}C NMR spectrum of PU-2.

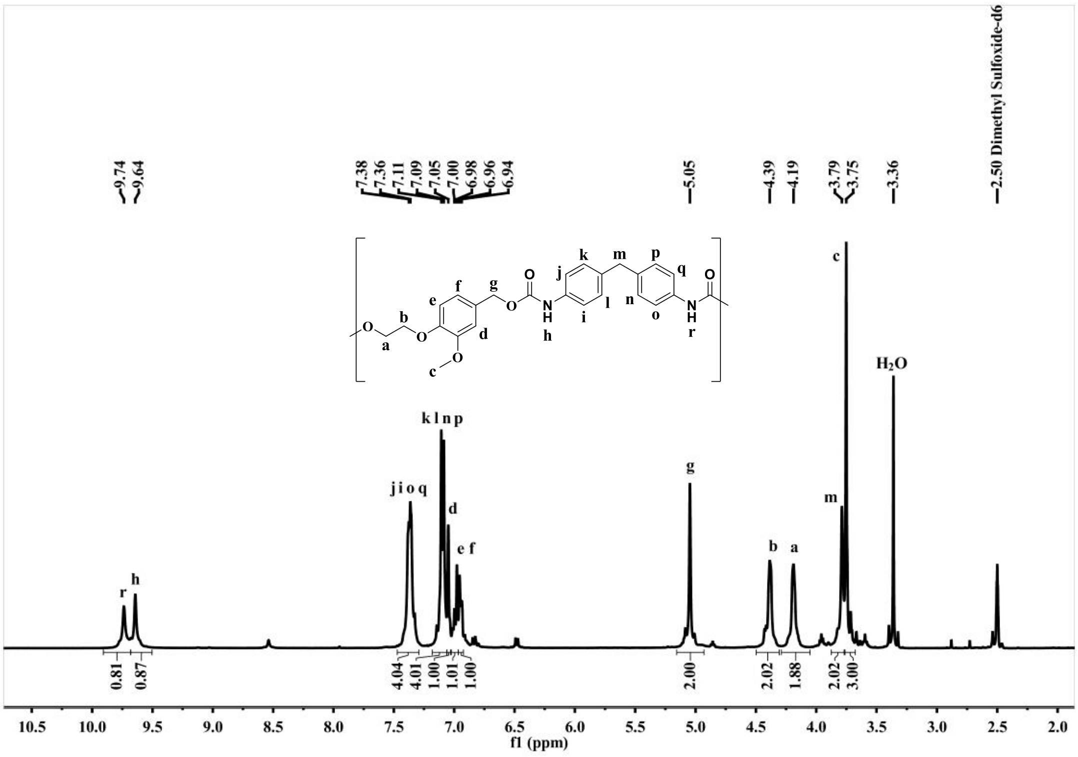

Figure S 19. ^{1}H NMR spectrum of PU-3.

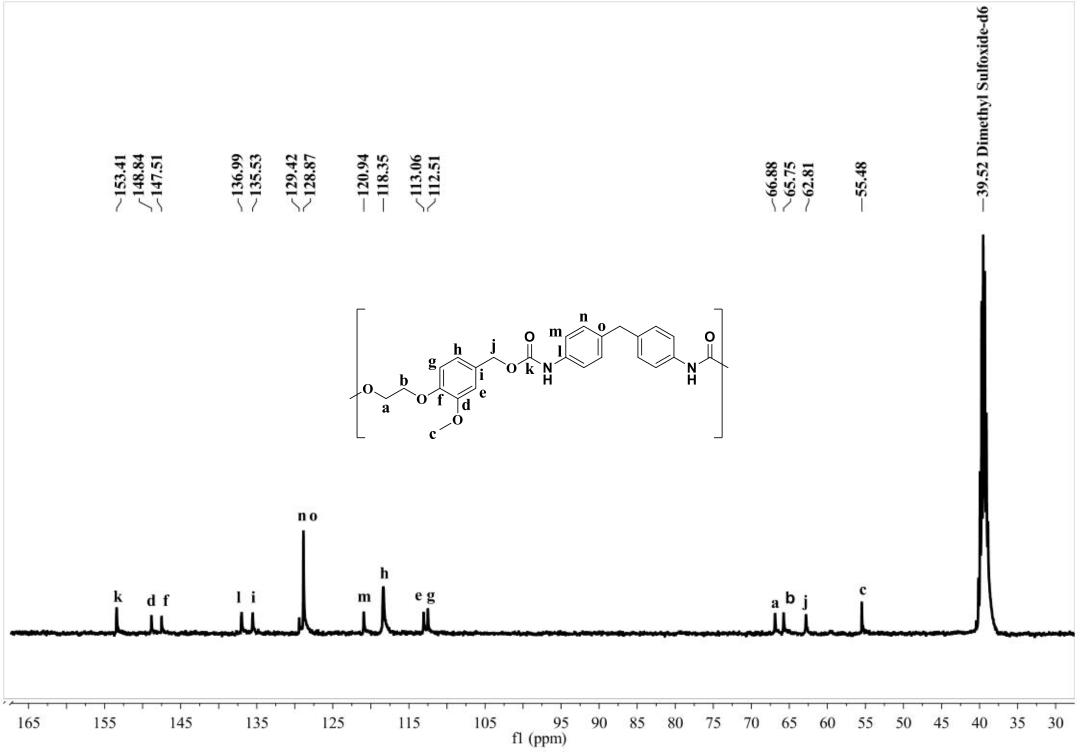

Figure S 20. ^{13}C NMR spectrum of PU-3.

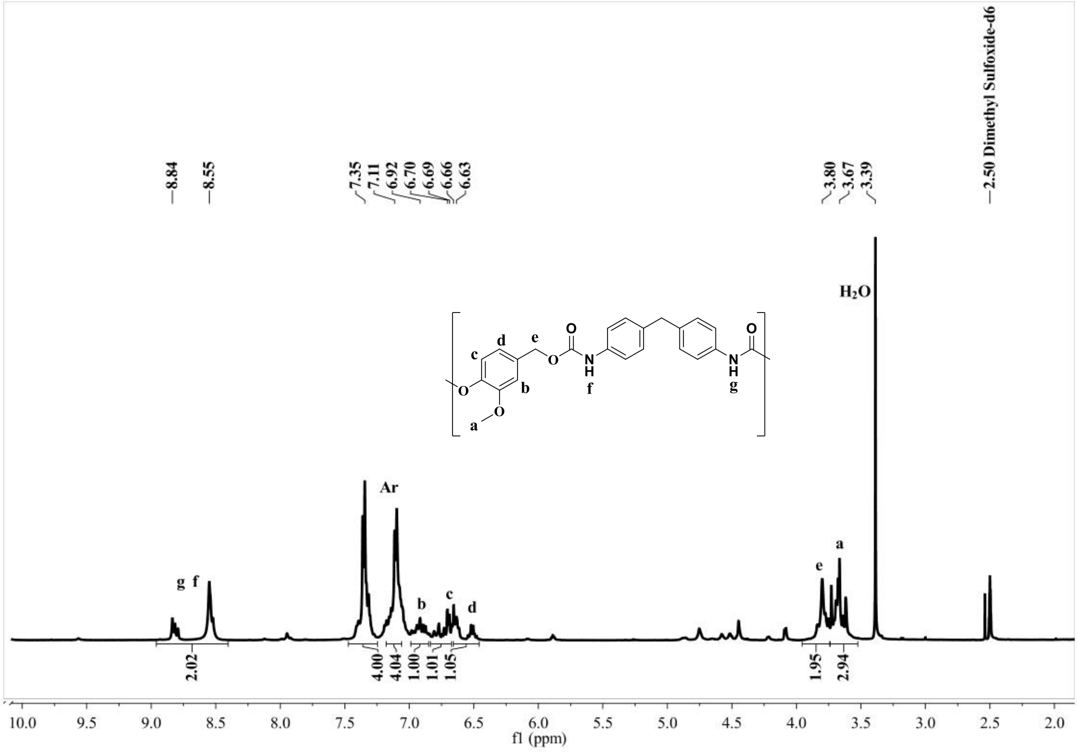

Figure S 21. ^{1}H NMR spectrum of PU-4.

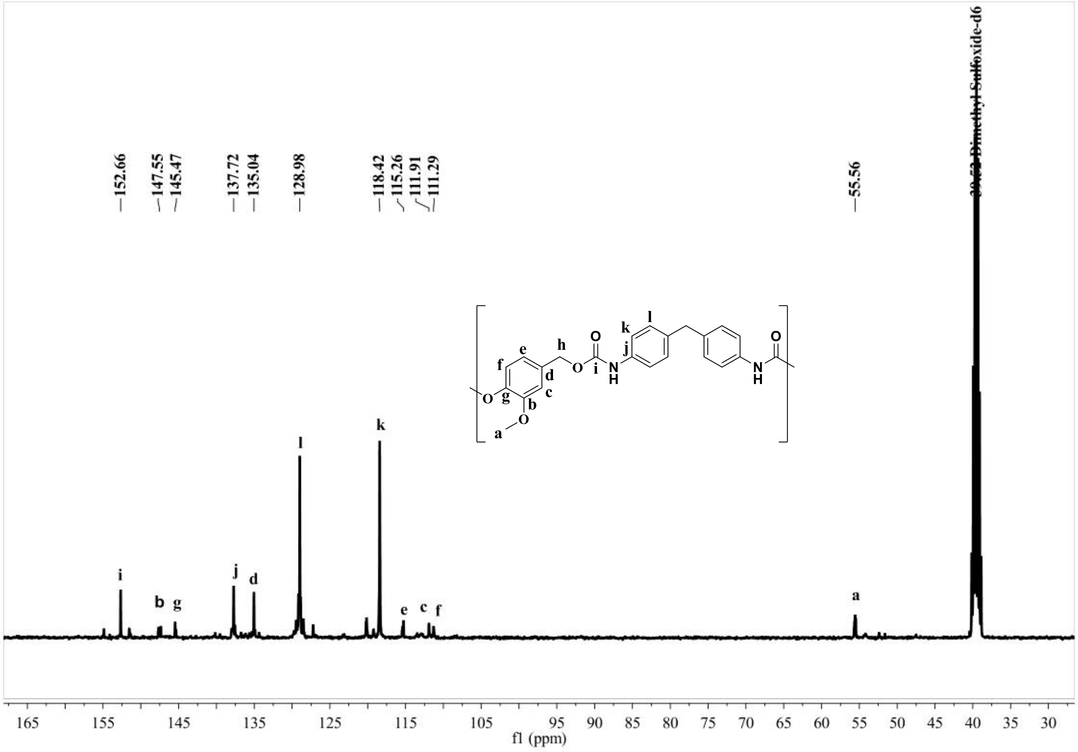

Figure S 22. ^{13}C NMR spectrum of PU-4.
